# Knowledge, attitudes, anxiety, and preventive behaviours towards COVID-19 among health care providers in Yemen: an online cross-sectional survey

**DOI:** 10.1186/s12889-020-09644-y

**Published:** 2020-10-13

**Authors:** Gamil Ghaleb Alrubaiee, Talal Ali Hussein Al-Qalah, Mohammed Sadeg A. Al-Aawar

**Affiliations:** 1 Department of Community Health, Faculty of Medical Sciences, Al-Razi University, Sana’a, Yemen; 2 Department of Applied Medical Sciences, Faculty of Medical Sciences, Al-Razi University, Sana’a, Yemen; 3 Department of Medical Laboratory, Faculty of Medical Sciences, Al-Razi University, Sana’a, Yemen

**Keywords:** SARS-CoV-2, COVID-19, Knowledge, Attitude, Anxiety, Preventive behaviours, Healthcare providers

## Abstract

**Background:**

The growing incidence of coronavirus (COVID-19) continues to cause fear, anxiety, and panic amongst the community, especially for healthcare providers (HCPs), as the most vulnerable group at risk of contracting this new SARS-CoV-2 infection. To protect and enhance the ability of HCPs to perform their role in responding to COVID-19, healthcare authorities must help to alleviate the level of stress and anxiety amongst HCPs and the community. This will improve the knowledge, attitude and practice towards COVID-19, especially for HCPs. In addition, authorities need to comply in treating this virus by implementing control measures and other precautions. This study explores the knowledge, attitude, anxiety, and preventive behaviours among Yemeni HCPs towards COVID-19.

**Methods:**

A descriptive, web-based-cross-sectional study was conducted among 1231 Yemeni HCPs. The COVID-19 related questionnaire was designed using Google forms where the responses were coded and analysed using the Statistical Package for the Social Sciences software package (IBM SPSS), version 22.0. Descriptive statistics and Pearson’s correlation coefficient test were also employed in this study. A *p*-value of < 0.05 with a 95% confidence interval was considered as statistically significant. The data collection phase commenced on 22nd April 2020, at 6 pm and finished on 26th April 2020 at 11 am.

**Results:**

The results indicated that from the 1231 HCPs participating in this study, 61.6% were male, and 67% were aged between 20 and 30 years with a mean age of 29.29 ± 6.75. Most (86%) held a bachelor’s degree or above having at least 10 years of work experience or less (88.1%). However, while 57.1% of the respondents obtained their information via social networks and news media, a further 60.0% had never attended lectures/discussions about COVID-19. The results further revealed that the majority of respondents had adequate knowledge, optimistic attitude, moderate level of anxiety, and high-performance in preventive behaviours, 69.8, 85.10%, 51.0 and 87.70%, respectively, towards COVID-19.

**Conclusion:**

Although the Yemeni HCPs exhibited an adequate level of knowledge, optimistic attitude, moderate level of anxiety, and high-performance in preventive behaviours toward COVID-19, the results highlighted gaps, particularly in their knowledge and attitude towards COVID-19.

## Background

A cluster of pneumonia cases of unknown origin or causes was reported in Wuhan, China, on 12th December 2019 [1]. Among the initial 41 cases reported, most originated from vendors and dealers working in the Huanan Seafood Market in Wuhan [2]. The World Health Organisation (WHO) and the Chinese authorities identified the causative agent as a new strain of coronavirus (SARS-CoV-2), named at that time as a coronavirus disease 2019, commonly referred to after that as COVID-19 [3]. Initially, SARS-CoV-2 quickly spread within China before dramatically spreading to other countries on a global scale [4]. On 11th March 2020, WHO declared the outbreak of COVID-19 as a global pandemic [5]. Since 12th September 2020, the virus has infected over 28,329,790 people, causing 911,877 deaths in 216 countries worldwide [6].

In Yemen, the fight against COVID-19 began on 10th April 2020 resulting from the initial case confirmed in Ash Shihr, the Hadramout province, southern Yemen. On 29th April 2020, five more cases of COVID-19 were confirmed and registered in Aden city, the temporary capital of Yemen. After that, the cases started to increase in other cities daily. Since 12th September 2020, 2011 cases of COVID-19 have been reported in the Republic of Yemen, of which 1211 cases have since recovered, resulting in 583 deaths. However, the number of COVID-19 cases is anticipated to be much higher than these figures, particularly given the transparency and the inability to effectively track and control the spread and number of cases reported in North Yemen [6].

At present, the exact dynamics and transmission of the virus have not been determined. However, according to WHO, the virus can be transmitted via air-droplets and fomites during close and unprotected contact between an infected person and a healthy person [7]. According to the Centre for Disease Control and Prevention (CDC) SARS-CoV-2 is transmitted from person to person through close contact (within 6 ft); from an infected person via respiratory droplets during coughing or sneezing or when touching a surface or an object that is contaminated with the virus, including touching one’s eyes, nose or mouth [8]. In most cases, those infected with COVID-19 experience none or mild to moderate symptoms that are alleviated within several weeks of isolation. However, in contrast, it can cause severe respiratory syndrome or death, particularly in older people or patients with chronic health diseases [9].

Similarly, healthcare providers (HCPs) as the front line defence in treating patients with COVID-19 are more susceptible to this spreading infection [10]. The WHO on 27th July 2020, estimated that close to 10% of all COVID-19 cases globally, which accounts for nearly 1.5 million cases, were related to HCPs. However, this figure is possibly underestimated, as, at that time, no systematic reporting or other measures were in place [11]. Indeed, information released by the International Council of Nurses (ICN), reported that until June 2020, nearly 230,000 HCPs worldwide had acquired COVID-19, with over 600 nurses dying [12].

In the context of Yemen, at present, the ongoing war and civil unrest over the past six years within the country has severely impacted or destroyed the much of the country’s infrastructure, with only 51% of the country’s health facilities remaining in operation [13]. This consists of two testing centres and 500 ventilators for a population of nearly 30 million people. Further, the country continues to suffer from limited testing capacity, critical shortage in health care supplies, including basic personal protective equipment (PPE) and other measures, limited by the ability to track the spread of the virus, especially, given the similarity COVID-19 symptoms with other diseases that already prevail in the country [14]. All these factors place the country sadly in a unique if not, an uncompromising and dangerous position should COVID-19 spread uncontrollably within the community, adding further burdening HCPs’ in the country.

However, viewing this situation from a wider perspective, the rapid spread of COVID-19 globally has caused considerable level anxiety, fear and panic among the population in countries worldwide, especially given that fact that HCPs and the elderly are most vulnerable to the risk of infection [15]. According to WHO, the shortage of appropriate PPE and other preventive measures directly endangers HCPs and represents a major cause of concern for countries [16]. Likewise, the availability and correct use of PPE is critical in order to protect and safeguard frontline workers such as HCPs in coping with COVID-19.

Though, what is of prime importance at this stage, is for HCPs to adhere to applying these preventive measures, which largely depend on their knowledge, attitude, and practice in addressing this highly contagious virus [2]. Nevertheless, Yemeni HCPs have been facing a double battle even before this pandemic eventuated given that Yemen, according to WHO, is more than 50% below the basic health services global benchmark concerning the coverage of health care services. Furthermore, while there are a limited number of skilled HCPs in the country, they have not received salaries for nearly five years. Surprisingly, the proportion of medics in Yemen has been calculated as 10 medics to every 10,000 of the population, notwithstanding that the number of nurses and midwives that are available remains inadequate to fill this shortage. These issues are further compounded by the ‘brain drain’ in the country of people seeking better opportunities offshore and weakening medical health education [17].

Therefore, to ensure the protection of HCPs and safeguard Yemen from COVID-19, there is an urgent need to upskill and enhance the understanding and awareness of COVID-19 among HCPs. This study aims to assess the knowledge, attitude, fear, and anxiety, as well as the preventive behaviours of HCPs towards COVID-19.

## Method

### Study area, study design, and study period

A descriptive, web-based cross-sectional survey was conducted among Yemeni HCPs between 22nd April 2020, 6 pm and 26th April 2020, 11 am. All HCPs who provided direct healthcare services to patients were invited to participate in the study.

### Study instrument

The questionnaire developed and used in this study was adapted from previously published studies based on the authors’ permission [2, 18]. The questionnaire consisted of 58 items that sought to collect information on the respondents’ knowledge, attitude, anxiety, and preventive behaviours toward COVID-19. The questionnaire comprised of five parts. Part (1) the socio-demographic characteristics such as age, sex, occupation, education level, years of working experience, and sources of COVID-19 related knowledge. Part (2) the respondents’ knowledge (21-items). Part (3) the respondents’ attitude (10-items) and Part (4) the respondents’ anxiety (17-items). Part (5) included questions on the respondents’ preventive behaviours (10-items).

### Scoring of knowledge, attitude, anxiety, and preventive behaviours

The scoring system that was used in this study was adapted from the work of Taghrir et al. [18] and Roy et al. [2]. The 21-items related to knowledge were assessed with either a “Yes” or “No” response in which each correct response was awarded a score of one (1), while a zero (0) score was assigned to an incorrect response. The scores ranged between 0 (no correct answers) and 21 (all answers are correct). A score of less than 11 was considered as having inadequate knowledge, and between 11 and 16, the scores were considered as having moderate knowledge, while a score of 17 and above was considered as having adequate knowledge.

Similarly, the 10-items signifying the respondents’ attitudes were evaluated with a “Correct” or “Incorrect” response. The scores ranging between zero (0) and seven (7) were considered as acquiring a negative attitude, while the scores between eight (8) and ten (10) were considered as having a positive attitude. The 17-items related to anxiety were assessed via a 5-point Likert scale, in which a score between 1 = “never” to 5 = “always”. The total cumulative score ranged between 17 and 85. Here, scores between 17 and 50 were considered as “low anxiety”, and those scores ranging between 51 and 67 were considered as “moderate anxiety”, while those ranging between 68 and 85 were considered as “high anxiety”. The 10-items related to preventive behaviours were assessed with a “Yes” or “No” response. A score between zero (0) and seven (7) was considered as “low performance”, while a score between eight (8) and ten (10) was considered as “high-performance”.

### Validity and reliability

Three experts with a background in infectious disease and epidemiology (one specialist in infectious disease and two epidemiologists) were invited to participate in assessing the content validity of the questionnaire items. The reliability of the questionnaire items was based on a pilot study that included 40 participants, and the reliability was tested using a Cronbach’s alpha test with the results showing 0.79 for the knowledge part, 0.77 for the attitude part, 0.80 for the anxiety part, and 0.75 for the preventive behaviours part.

### Data collection

At present, due to the outbreak of COVID-19 and the specific preventive precautions and measures recommended by the Ministry of Health and Population in Yemen, an electronic web-based self-reported questionnaire was designed to comply with the recommendations. The internet link was distributed to the HCPs via email, WhatsApp, Telegram, and other forms of social media. The criteria of the HCPs to participated in the study needed to be living in the Republic of Yemen, regardless of gender, aged 20 years or older, was aware of the COVID-19 outbreak, and who had signed a consent form to participate in the study. Although participation in the study was voluntary, personal details of the participants were not recorded on the questionnaire. The respondents in receipt of the questionnaire were encouraged to forward the survey to other colleagues who may be interested in participating in the study as well.

### Ethical consideration

Approval of the Ethics Committee of Al-Razi University was obtained before conducting the study. The respondents needed to confirm their willingness to participate on a voluntary basis by answering a “Yes or No” question on a written informed consent form before being allowed to complete the online self-reporting questionnaire.

### Data analysis

The Statistical Package for Social Sciences (IBM SPSS), version 22.0 was used in the administration and analysis of the collected data. Descriptive analyses using mean values and standard deviations for continuous variables and the count and percentages for the dichotomous or categorical variables were used in describing the data. The relationship between the study variables was assessed using Pearson’s correlation coefficient test. A *p-*value of < 0.05 (two-tailed) with a 95% confidence interval was reported as significant for the correlation analysis.

## Results

### Healthcare providers’ demographic characteristics

The respondents’ socio-demographic data are presented in Table 1 below. As shown in the table, over half (61.6%) of the HCPs were male, with more than (67%) of respondents were aged between 20 and 30 years with a mean of 29.29 ± 6.75. Regarding the occupation of respondents, 22.5% were physicians, followed by pharmacists (17.8%), laboratory technicians/workers (16.5%), and nurses (16.0%). Regarding their education and working experience, 4.5% of respondents held a PhD, 1.8% held a Board position with 88.1% of all respondents having 10 years or less of working experience. Concerning COVID-19 related information sources, social media was highlighted as the main source (31.0%) followed by news media (26.1%). Around 99.0% of respondents were aware of COVID-19, with a further 60.0% having never attended lectures or discussions on COVID-19.

**Table 1:** Healthcare providers’ demographic characteristics

Demographic characteristic		no(%)
Age	20–30	825 (67)
	31–40	313 (25.4)
	41–50	79 (6.4)
	51–60	14 (1.1)
	Mean ± SD	29.29 ± 6.75
Sex	Male	758 (61.6)
	Female	473 (38.4)
Occupation	Physician	277 (22.5)
	Nurses	197 (16.0)
	Laboratory	203 (16.5)
	Anesthesia	55 (4.5)
	Dentist	53 (4.3)
	Medical Academicians	72 (5.8)
	Pharmacist	219 (17.8)
	Community	55 (4.5)
	Midwifery	25 (2.0)
	Physiotherapy	20 (1.6)
	Nutrition	45 (3.7)
	Radiology	10 (0.8)
Education Level	Diploma	172 (14.0)
	Bachelors	899 (73.0)
	Master	82 (6.7)
	Ph.D	56 (4.5)
	Board	22 (1.8)
Experience Years	0–10	1084 (88.1)
	11–20	125 (10.2)
	> 20	22 (1.8)
	Mean ± SD	4.25 ± 5.59
Source of COVID-19 data	News media	712 (26.1)
	Social media	846 (31.0)
	Ministry of health and WHO	545 (20.0)
	Family and friend	302 (11.1)
	Working Place	321 (11.8)
Heard about Novel COVID-19	Yes	1231 (99.0)
Attended lectures/discussions about novel COVID-19	Yes	492 (40.0)
	No	739 (60.0)

### Healthcare providers’ level of knowledge regarding the COVID-19 pandemic

**Fig. 1:**
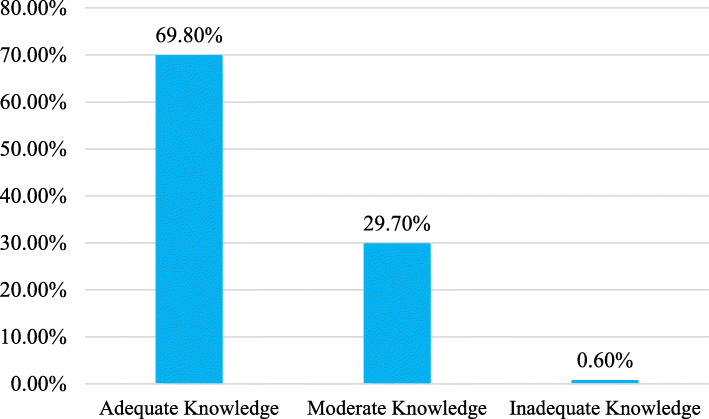
Healthcare providers’ level of knowledge on COVID-19 pandemic

The level of knowledge among healthcare providers regarding the COVID-19 pandemic is presented in Fig. 1 below. Twenty-one items within the questionnaire instrument having a “True” or “False” response choice was used to assess the extent of the respondents’ knowledge regarding COVID-19. As shown in Fig. 1, the majority of HCPs (69.80%) were believed to have acquired an adequate level of knowledge regarding COVID-19, while 29.70% had moderate knowledge, and only 0.60% were considered to have inadequate knowledge. The lower percentages were attributed to four (4) statements that discussed the importance of wearing face masks, the need to wear N95 face masks only during intubation, suction, bronchoscopy, and cardiopulmonary resuscitation, in treating the disease by usual antiviral drugs and antibiotics as the first-line (of defence) treatment, that scored 69.9, 68.8, 28.47, and 27.3%) respectively.

### Healthcare providers’ attitude towards the COVID-19 pandemic

**Fig. 2:**
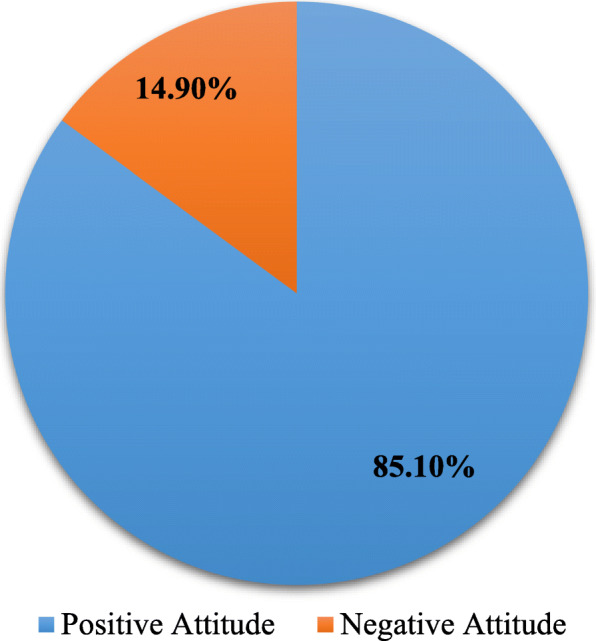
Healthcare providers’ attitude toward COVID-19 pandemic

The level of attitude of Yemeni HCPs towards the COVID-19 pandemic is shown in Fig. 2 below. The respondents’ attitude towards the COVID-19 pandemic was assessed using ten (10) items with a “Yes” or “No” response choice. As shown in Fig. 2, the findings indicate that the majority of respondents (85.10%) had a positive attitude, while 14.90% had a negative attitude towards the COVID-19 pandemic. However, although the vast majority of respondents exhibited a high degree of optimism and attitude towards the pandemic, 75.1% still believed that they would not contract the disease, and almost 29.4% were willing to move to other locations within the country to be safe and secure during the pandemic.

### Healthcare providers’ anxiety toward the COVID-19 pandemic

**Fig. 3:**
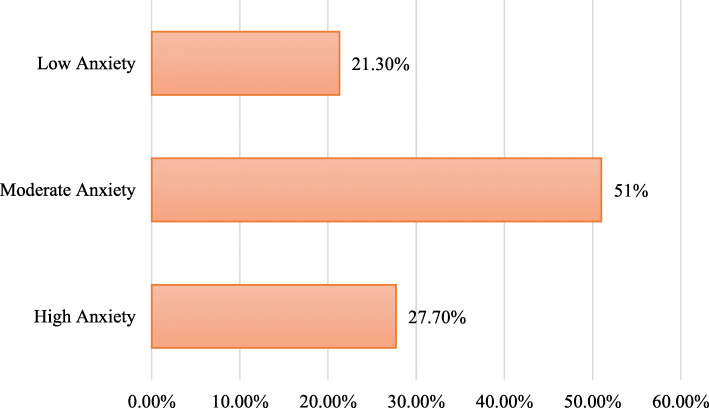
Healthcare providers’ anxiety of COVID-19 pandemic

The level of anxiety among Yemeni HCPs toward the COVID-19 pandemic is illustrated in Fig. 3 below. The level of anxiety among HCPs was assessed using 17-items, with the answers rated against a 5-point Likert ranging between 0 = “never” to 5 = “always”. As shown in Fig. 3, the findings indicate that just of half of the respondents had a moderate level of anxiety towards the pandemic, 27.70% had a high level of anxiety, and 21.30% had a low level of anxiety towards the COVID-19 pandemic.

### Healthcare providers’ self-reported preventive behaviours toward the COVID-19 pandemic

**Fig. 4:**
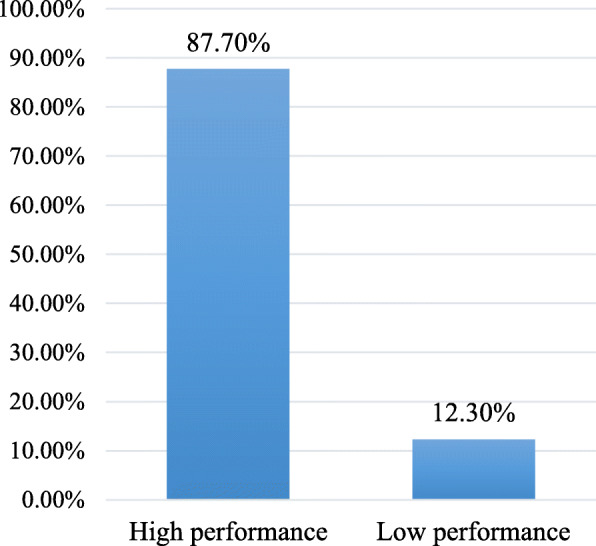
Healthcare providers’ preventive behaviours toward COVID-19 pandemic

Ten-items each requiring a “Yes” or “No” response was used to assess the respondents’ level of self-reported preventive behaviours towards COVID-19. Five (5) items were to avoid or reduce visiting public places in their daily life. One item was related to preventive behaviour such as coughing/sneezing, two items were related to hand washing and frequently disinfecting surface areas on a frequent basis, and one item was related to talking with family and friends about preventive measures associated with of COVID-19. As can be seen in Fig. 4, the vast majority (87.70%) of respondents exhibited sufficient preventive behaviours, while only 12.30% demonstrated low preventive behaviours. The lowest score (84.8%) was related to cancelled or postponed activities and events such as eating out, sporting activities, and meeting with colleagues.

### Association between the respondents’ socio-demographic characteristics and their knowledge, attitude, anxiety, and preventive behaviours

The association between the respondents’ socio-demographic characteristics and their knowledge, attitude, anxiety, and preventive behaviours towards the COVID-19 pandemic are reflected in Table 2 below. As can be seen in the table, the results reveal that there was a significant positive correlation between the respondents’ level of knowledge of COVID-19 and their occupation and education level with a *p*-value of 0.016 and 0.001, respectively. Further, there was a significant positive correlation between the respondents’ level of attitude towards COVID-19 and their occupation, with a *p*-value of 0.018. Moreover, there was a significant positive association between the respondents’ anxiety and their gender and educational level with a *p*-value of 0.014 and 0.004, respectively. Regarding the preventive behaviours towards COVID-19, there was a significant positive association between the respondents’ level of performance and their gender, occupation, years of work experience, and educational level with a *p*-value of 0.010, 0.023, 0.011 and 0.001, respectively.

**Table 2:** Association between the respondents’ socio-demographic characteristics, Knowledge, attitude, anxiety and preventive behaviours

Demographic characteristic		Knowledge	Attitudes	Anxiety	Preventive behaviors
Sex	Male	17.18 ± 1.99	8.62 ± 1.24	59.33 ± 12.53	9.02 ± 1.70
	Female	16.96 ± 2.00	8.66 ± 1.28	61.10 ± 12.08	9.27 ± 1.51
	***p*** **value**	**0.058**	**0.585**	**0.014**	**0.010**
Age (years)	18–30	17.22 ± 1.83	8.63 ± 1.28	60.58 ± 12.39	9.11 ± 1.61
	31–40	16.77 ± 2.42	8.63 ± 1.16	59.13 ± 13.02	9.06 ± 1.86
	41–50	17.17 ± 1.79	8.68 ± 1.30	57.88 ± 10.01	9.30 ± 1.06
	51–60	16.57 ± 0.75	9.07 ± 1.20	58.64 ± 6.60	9.85 ± 0.36
	***p*** **value**	**0.005**	**0.622**	**0.120**	**0.246**
Occupation	Physician	17.23 ± 1.50	8.79 ± 1.31	59.674 ± 11.22	9.36 ± 1.28
	Nurses	16.77 ± 2.73	8.63 ± 1.28	60.34 ± 12.28	8.95 ± 1.77
	Laboratory	17.14 ± 2.34	8.68 ± 1.06	61.24 ± 13.86	8.97 ± 1.91
	Anesthesia	17.20 ± 2.04	8.56 ± 0.83	62.20 ± 11.14	9.52 ± 0.79
	Dentist	16.60 ± 1.81	8.56 ± 1.46	59.73 ± 11.97	9.57 ± 0.79
	Academics	17.08 ± 1.45	8.93 ± 1.12	59.38 ± 12.49	8.97 ± 1.56
	Pharmacist	17.35 ± 1.75	8.47 ± 1.22	59.29 ± 12.43	9.04 ± 1.81
	Community	17.01 ± 1.52	8.20 ± 1.71	56.72 ± 13.78	8.72 ± 2.11
	Midwifery	16.44 ± 2.38	8.36 ± 1.38	56.20 ± 10.70	9.32 ± 1.06
	Physiotherapy	17.00 ± 1.62	8.80 ± 1.10	59.75 ± 13.36	9.05 ± 1.27
	Nutrition	17.00 ± 1.39	8.82 ± 1.09	63.22 ± 12.46	8.97 ± 2.05
	Radiology	18.70 ± 1.41	8.20 ± 1.03	62.80 ± 10.65	9.40 ± 1.26
	***p*** **value**	**0.016**	**0.018**	**0.191**	**0.023**
Years of working experience	≤10	17.11 ± 2.04	8.64 ± 1.25	60.23 ± 12.71	9.06 ± 1.70
	11–20	16.96 ± 1.72	8.56 ± 1.24	58.88 ± 9.03	9.51 ± 0.95
	> 20	17.22 ± 1.34	9.05 ± 1.21	55.59 ± 11.53	9.45 ± 1.22
	***p*** **value**	**0.705**	**0.256**	**0.123**	**0.011**
Educational Level	Diploma	17.02 ± 2.95	8.59 ± 1.38	58.77 ± 12.54	8.95 ± 1.79
	Bachelors	17.19 ± 1.70	8.66 ± 1.22	60.47 ± 12.40	9.11 ± 1.61
	Master	17.21 ± 1.39	8.64 ± 1.07	59.56 ± 10.70	9.62 ± 0.76
	Ph.D.	15.85 ± 3.13	8.39 ± 1.55	55.12 ± 13.63	8.62 ± 2.40
	Board	16.77 ± 1.34	8.63 ± 1.17	65.09 ± 8.81	9.90 ± 0.42
	***p*** **value**	**0.001**	**0.593**	**0.004**	**0.001**

### Correlation between respondents’ knowledge, attitude, anxiety, and preventive behaviours scores

The correlation between HCPs knowledge, attitude, anxiety, and preventive behaviour scores is shown in Table 3 below. The correlations were divided into four (4) levels based on the following criteria: weak = 0–0.25, fair = 0.25–0.5, good = 0.5–0.75, and excellent = 0.75 or greater [19]. As shown in Table 3, there was a significant positive linear correlation between knowledge-attitude (r = 0.176, *p* < 0.001), knowledge-anxiety (r = 0.136, *p* < 0.001), knowledge-preventive behaviours (r = 0.320, *p* < 0.001), attitude-anxiety (r = 0.078, *p* < 0.006), attitude-preventive behaviours (r = 0.293, *p* < 0.001) and anxiety-preventive behaviours (r = 0.284, *p* < 0.001). Accordingly, the results indicate the relationship between knowledge, attitude, anxiety, and preventive behaviours towards the COVID-19 pandemic.

**Table 3:** Correlation between respondents’ knowledge, attitude, anxiety and preventive behaviours scores

Variable	Correlation coefficient	***P***-value
Knowledge-Attitude	0.156	0.001**
Knowledge-Anxiety	0.136	0.001**
Knowledge-Preventive behaviors	0.320	0.001**
Attitude-Anxiety	0.078	0.006**
Attitude-Preventive behaviors	0.293	0.001**
Anxiety-Preventive behaviors	0.284	0.001**

**. Correlation is significant at the 0.01 level (2-tailed)

## Discussion

Since the first confirmed case announced in Yemen on 10th April 2020, in Ash Shihr, (a port city in the Hadhramaut Province of southern Yemen), rising fear and anxiety extended to other provinces from the possibility of contracting COVID-19 and its outbreak. The HCPs as the front line of defence and older people were the most vulnerable in contracting COVID-19 that the majority of other people. During this time, there was also a critical shortage of PPE given the current conflict in the region, and civil unrest in the country [14]. Equally important was the need during this period to understand the level of preparedness of HCPs’ in order to cope with the outbreak of COVID-19 in the country. This fact motivated the need to undertake the current study aiming to explore the level of knowledge, attitude, anxiety, and preventive behaviours among HCPs towards the outbreak of COVID-19 in the country.

The findings have shown that while the majority of respondents (60.0%) had never attended COVID-19 training courses with respect to COVID-19, most (69.80%) had acquired an adequate level of knowledge about the outbreak of the virus. On the other hand, the four (4) statements reflecting the importance of wearing face masks in the community, having to wear N95 face masks only during intubation, suction, bronchoscopy, and cardiopulmonary resuscitation, the possibility to treat the disease using antiviral drugs and antibiotics as first-line treatment scored the lowest at 69.9, 68.8 28.47 and 27.3%, respectively. This result possibly highlights the need to direct more attention toward developing educational courses and programmes related to COVID-19.

Likewise, the adequate level of knowledge among the respondents could be attributed to their educational level since most (73.0%) of respondents held a bachelor’s degree or higher, (i.e. a master’s degree). Accordingly, an educated professional group such as this could help to collect knowledge of COVID-19 from a variety of information sources. Moreover, the results also showed that only (20.0%) of HCPs gained information about COVID-19 from the official websites of the Ministry of Public Health and Population and WHO. This suggests that health authorities should direct more attention towards encouraging HCPs to use official websites as an essential and credible source of information about COVID-19. Likewise, 57.1% of HCPs seemed to use social media and news media as the main source of information, which is a significant concern given the reliability of this information. This is because utilising such media can mislead HCPs by spreading fabricated and unverified information. It is also worth highlighting that the respondents’ level of knowledge was only statistically significantly different according to their age, occupation, and educational level.

Furthermore, these results are consistent with the results of a previous study [20] which reported that the level of knowledge towards COVID-19 differs significantly across different age groups, educational levels, and levels of different professions. The results are also in line with the results of Giao et al. [9] and Saqlain et al. [21] regarding the difference in the level of respondents’ anxiety based on their profession. Concerning the level of respondents’ attitude, it was found to differ based on the participants’ occupations significantly. This corroborates with a study by Giao et al. [9], which reported a significant association between respondents’ attitude and their occupation.

However, in contrast, the result seems in differ from the results of Saqlain et al. [21] and Rahman and Sathi [20], who stated that a positive attitude toward COVID-19 did not significantly vary nor differ across different occupations. Equally, the results revealed that the respondents’ level of anxiety was significantly different based on their gender and educational levels. These results support the findings reported by Al-Hanawi et al. [22] that respondents’ level of worry or concern attributed to COVID-19 differs significantly across gender and educational level. This result is also in line with previous studies [23, 24] carried out in China, indicating that females have higher levels of anxiety compared to males.

Similarly, the respondents’ level of self-reported preventive behaviour significantly differed according to their gender, occupation, years of working experience, and educational level. These results are in agreement with the results by Rahman and Sathi [20] on the variation of respondents’ preventive behaviour according to different age groups, Al-Hanawi et al. [25] regarding the gender of respondents, Saqlain et al. [21] regarding the respondents’ years of working experience and Khasawneh et al. [26] about the respondents’ educational level.

With respect to the attitude of the respondents’, the result showed that 85.10% of respondents had an optimistic attitude towards COVID-19, though unfortunately, the findings also revealed that 75.1% believe that they avoid infection, and close to 29.4% of respondents were willing to relocate to protect themselves from COVID-19. This result suggests that most of the respondents were either confident of protecting themself from the virus or unaware about the nature of COVID-19 how contagious it is. Similarly, one-third of respondents would look to leave their work and relocate for fear of infection, which contributed to the shortage of HCPs if the situation was to become more serious, i.e. rising infections.

Accordingly, based on the results and the information presented above, it is imperative given the seriousness of the issue that training courses and awareness programmes be created on COVID-19 and disseminating such information via official websites. Regarding the high level of optimism and attitude of respondents in the current study, this could also be explained, at this stage, by the limited number of cases reported in Yemen, and the adequate level of knowledge they had gained since the outbreak of the virus, and until this research study was conducted.

According to Roy et al. [2], adequate awareness often leads to optimistic attitudes, which could positively affect the preparedness of HCPs to address pandemic issues. Furthermore, the results of the current study showed a positive correlation between the respondents’ knowledge and their attitude, which could support this conjecture. Moreover, the findings of the current study are consistent with a study by Giao et al. [9], that healthcare workers had a high level of knowledge and a positive attitude towards COVID-19. These findings are also in line with the results of a cross-sectional study conducted among Saudi health college students [27], which revealed that more than half of the students had a positive attitude towards MERS-CoV.

Concerning the respondents’ level of anxiety, the results indicated that nearly half (51%) of the respondents had a moderate level of anxiety and 27.70% had a high level of anxiety regarding the COVID-19 outbreak. According to Roy et al. [2], fear and anxiety within a population are usually expected given the significant impact of the pandemic on the community, which could also affect the mental well-being of people and influence their behaviour in the wider community. In this study, only 27.7% of the respondents exhibited a high level of anxiety concerning COVID-19, which could possibly be attributed to their level of knowledge given they were still experiencing the first wave of the virus COVID-19. Interesting, the current study indicated lower anxiety level results compared to other studies that were carried out during the outbreak as reported by Huang and Zhao [28] on Chinese healthcare workers and Nemati et al. [29] on Iranian nurses. In these studies, the results showed that the level of anxiety among healthcare workers was higher compared to other people. The high anxiety level among the HCPs could be attributed to the uncontrolled nature of the pandemic and concerns of becoming infected, particularly given the shortage of healthcare institutions and PPE.

Concerning the self-reported preventive behaviours, it was found that the majority (87.70%) of respondents had a high-performance level of preventive behaviours towards COVID-19, which could be attributed to the having an adequate level of knowledge and awareness among the respondents towards COVID-19. As reported in a previous study, those who had acquired adequate knowledge exhibited optimistic attitudes and appropriate, it not proactive practices toward COVID-19 [30]. Another study revealed that the level of good or sound knowledge in a given population about COVID-19 is significantly reflected in their behaviour and attitude [2].

However, the findings from the current study were seemingly lower than a study conducted during COVID-19 by Taghrir et al. [18] on medical students in Iran finding that 94.2% of the respondents showed relatively high-performance in preventive behaviours toward COVID-19. According to the results of this study, females were found to exhibit a higher-performance-level in preventive behaviours compared to their male counterparts, possibly due to their better compliance in preventive measures towards COVID-19. This result is consistent with the result by Taghrir et al. [18] that females demonstrated more precautionary behaviours compared to males.

Notwithstanding, another key result in this study was of the positive linear correlation between knowledge-attitude, knowledge-anxiety, knowledge-preventive behaviours, attitude-anxiety, attitude-preventive behaviours, and anxiety-preventive behaviours. This result confirms the relationship between the respondents’ level of knowledge and their level of anxiety, attitude, and preventive measures towards COVID-19. Such a correlation could be explained by the Theory of Reasoned Action (TRA) [31], which states that a person’s intention to carry out a specific behaviour is determined by their attitude towards this behaviour.

In the current study, the findings are in line with the results of other studies [20, 30, 32] showing that acquiring a good level of knowledge of COVID-19 is correlated with optimistic attitudes and proper practices towards COVID-19. However, in contrast, the results of this study disagree with the results by Nemati et al. [29] in which they found that most Iranian nurses displayed their anxiety and that of their families as a result of COVID-19 though the knowledge they had acquired about COVID-19 to be sufficient. Lin et al. [24] found that the level of knowledge of COVID-19 did not influence anxiety levels. However, they found that higher levels of attitude were highly associated with high levels of anxiety.

Furthermore, in a study carried out in Hong Kong by Leung et al. [33], the results revealed that the level of anxiety during the SARS outbreak was highly associated with behavioural responses such as wearing face masks. In a separate study by Roy et al. [2], they revealed that people’s level of anxiety correlated with their behaviour. The results showed that under the effect of rumours, people tend to modify their behaviour positively compared to an undesirable one. Reuben et al. [20] also reported the relationship between respondents’ attitudes and their preventive behaviours. Regarding the relationship between the respondents’ attitudes and their preventive behaviour, Rubin et al. [34] conducted a study during the swine flu outbreak, reporting a significant association between the respondents’ attitude and their behavioural change (e.g. performing one or more avoidance behaviours).

Nevertheless, several limitations were inherent in this study which should be addressed for future research. The first limitation concerns the nature of collecting the data. The data in this study were collected via a web-based survey since it was not possible to conduct a face-to-face survey among Yemeni HCPs during given the uncertainty surrounding the outbreak of the virus and level of contagious. Therefore, the data may be seen as being less reliable having less accountability compared to face-to-face interviews and the lack of a trained interviewer. Secondly, collecting the data was challenging, given the availability of respondents and cooperation. Thirdly, the exclusiveness of the study to HCPs. Therefore, future research should involve a more diverse community or population, employing a community-based study design.

## Conclusion

The results of this study have demonstrated that the majority of HCPs in Yemen had acquired an adequate level of knowledge of COVID-19. However, their level of knowledge concerning situations that require wearing N95 masks and the possibility of using current antiviral drugs and antibiotics as the first-line of treatment for COVID-19 could be improved through training and other programmes. The moderate anxiety level, as revealed in this study, would undoubtedly increase, particularly if the prevalence curve of the outbreak of COVID-19 elevated, and the situation became much worse. Therefore, implementing preventive measures and regulation strategies to control the emotional status among HCPs is recommended. In addition, organisations such as WHO and the Ministry of Public Health and Population in Yemen must continue to provide updated information regarding COVID-19 to warrant better control concerning COVID-19.

## Data Availability

Data are available from the corresponding author on a reasonable request.

## Abbreviations

COVID-19: Coronavirus Disease 2019
SARS-CoV-2: Severe acute respiratory syndrome coronavirus 2
HCPs: Healthcare providers
WHO: World Health Organisation
CDC: Centre for Disease Control and Prevention
IBM SPSS: Statistical Package for Social Sciences
